# Peroxisome: the new player in ferroptosis

**DOI:** 10.1038/s41392-020-00404-3

**Published:** 2020-11-24

**Authors:** Daolin Tang, Guido Kroemer

**Affiliations:** 1 Department of Surgery, University of Texas Southwestern Medical Center, Dallas, Texas 75390 USA; 2 Center for DAMP Biology, University of Texas Southwestern Medical Center, Dallas, Texas 75390 USA; 3 Equipe labellisée par la Ligue contre le cancer, Université de Paris, Sorbonne Université, INSERM U1138, Centre de Recherche des Cordeliers, Paris, France; 4 Metabolomics and Cell Biology Platforms, Gustave Roussy Cancer Campus, 94800 Villejuif, France; 5 Pôle de Biologie, Hôpital Européen Georges Pompidou, AP-HP, 75015 Paris, France

**Keywords:** Cell biology, Molecular biology

**Fig. 1:**
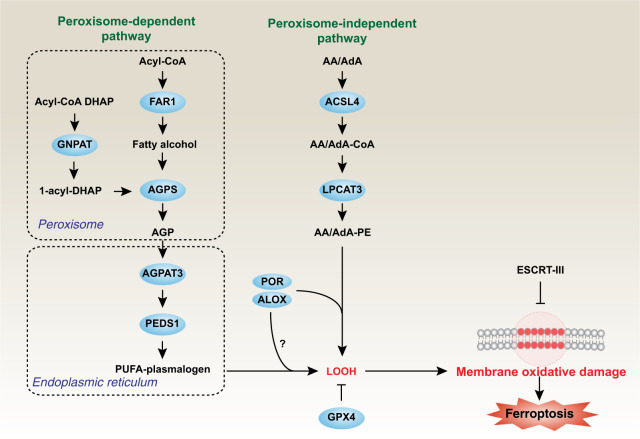
Peroxisome-dependent and independent initiation of ferroptotic pathways. The peroxidation of PUFAs by ALOXs or POR to produce LOOH is an important step in promoting ferroptosis. The synthesis of PUFA-plasmalogen and AA/AdA-PE is mediated by peroxisome-dependent and independent pathways, respectively. Ferroptosis is suppressed by the lipid peroxidation repair enzyme GPX4 and the ESCRT-III membrane repair machinery. AA/AdA arachidonic acid/adrenic acid, AA/AdA-CoA, arachidonic acid/adrenic acid-coenzyme A, AA/AdA-CoA-PE arachidonic acid/adrenic acid-coenzyme A-phosphatidylethanolamine, Ac-CoA acetyl coenzyme A, ACSL4 acyl-CoA synthetase long-chain family member 4, ALOX lipoxygenase, AGPS alkylglycerone phosphate synthase, AGP 1-O-alkyl-glycerol-3-phosphate, AGPAT3 1-acylglycerol-3-phosphate O-acyltransferase 3, DHAP dihydroxyacetone phosphate, ESCRT-III endosomal sorting complexes required for transport-III, FAR1 fatty acyl-CoA reductase 1, GPX4 glutathione peroxidase 4, GNPAT glyceronephosphate O-acyltransferase, LPCAT3 lysophosphatidylcholine acyltransferase 3, LOOH lipid hydroperoxides, POR cytochrome P450 oxidoreductase, PUFAs polyunsaturated fatty acids, PEDS1/TMEM189 plasmanylethanolamine desaturase 1

A recent paper published in *Nature* by Zou et al. reported that peroxisomes, membrane-bound oxidative organelles, contribute to ferroptosis through the biosynthesis of plasmalogens for lipid peroxidation (Fig. 1).^1^ These observations provide new insights into the lipid metabolic basis of ferroptotic cell death.

Cell death, which is essential for organismal homeostasis, exhibits multiple subroutines with different molecular mechanisms and signaling cascades.^2^ Within the expanding typology of regulated cell death pathways, ferroptosis is an iron-dependent non-apoptotic cell death caused by unrestrained lipid peroxidation culminating in irreversible plasma membrane damage.^3^ The basic process of ferroptosis involves the production of free radicals, the supply of fatty acids, and the increase in specialized enzymes (e.g., lipoxygenase [ALOX] and cytochrome P450 oxidoreductase [POR]) responsible for lipid peroxidation.^4^ More specifically, the oxidation of polyunsaturated fatty acids (PUFAs) appears to be a key driver of ferroptosis. Distinct types of PUFAs are associated with different risks of pathological conditions and diseases. Previous studies have shown that PUFAs are esterified into membrane phospholipids, especially phosphatidylethanolamine (PE)-containing phospholipids (arachidonic acid and adrenic acid), and then generate lipid peroxides, leading to ferroptosis in various cells or tissues.^5^ The acyl-CoA synthetase long-chain family member 4 (ACSL4)-lysophosphatidylcholine acyltransferase 3 (LPCAT3) pathway is responsible for the production of most PUFAs during ferroptosis. In contrast, the lipid peroxidation repair enzyme glutathione peroxidase 4 (GPX4) and the endosomal sorting complexes required for the transport (ESCRT)-III membrane repair machinery can prevent ferroptosis. Now, Zou et al.^1^ report that peroxisome-dependent plasmalogen production constitutes yet another, ACSL4/LPCAT3-independent phospholipid resource susceptible to ferroptotic oxidative damage in cancer cells, neurons, and cardiomyocytes (Fig. 1).

In a first step, the authors performed genome-wide CRISPR–Cas9 suppressor screens to identify new genes that regulate the susceptibility of two tumor cell lines to GPX4 inhibitor (RSL3 and ML210)-induced ferroptosis: the clear cell renal cell carcinoma 786-O cell line and the human ovarian carcinoma OVCAR-8 cell line. This procedure led to a marked enrichment of peroxisome-related genes, the deletion of which increased cellular viability. Peroxisomes play multiple roles, including the production or elimination of hydrogen peroxide, the synthesis of certain lipids, and the degradation of long-chain and branched-chain fatty acids. Through the depletion or overexpression of peroxisome biogenesis genes (such as peroxisomal biogenesis factor 10 [PEX10] and PEX3), the authors found that the number of peroxisomes was positively correlated with susceptibility to ferroptosis. Therefore, peroxisomes may be added to the list of organelles that can initiate the ferroptotic cell death.^5^

Subsequently, the author explored how peroxisomes affect the sensitivity of cells to ferroptosis. Peroxisomal enzymes involved in the synthesis of plasmalogens, such as alkylglycerone phosphate synthase (AGPS), fatty acyl-CoA reductase 1 (FAR1), and glyceronephosphate O-acyltransferase (GNPAT), were significantly enriched among the CRISPR targets that confer cytoprotection. Lipidomic analysis revealed that the production of plasmalogens was diminished in PEX3- or PEX10-deficient cells. These experiments indicate that peroxisomes affect the sensitivity of cells to ferroptosis by synthesizing plasmalogens, a subclass of ether phospholipids that are abundant in cell membranes in the cardiovascular, immune, and nervous systems. At difference with common phospholipids, plasmalogens use an ether (instead of an ester) bond to connect the glycerophospholipid. Their sn-1 chains are non-hydrolyzable ether-linked chains, while their sn-2 chains are connected by conventional ester bonds. Due to this special chemical structure, the synthesis of plasmalogen precursor needs to be carried out independently in the peroxisome. Here, the precursor 1-O-alkyl-glycerol-3-phosphate (AGP) is synthesized by FAR1 and AGPS and then transported to the endoplasmic reticulum (ER) for further biosynthetic reactions involving 1-acylglycerol-3-phosphate O-acyltransferase 3 (encoded by AGPAT3). Finally, the ER-resident enzyme plasmanylethanolamine desaturase 1 (PEDS1, also known as TMEM189) mediates the production of PUFA-plasmalogen. Importantly, the provision of liposomal nanoparticles composed of purified plasmalogens to cells was sufficient to confer sensitivity to ferroptosis by lipid peroxidation, further confirming the implication of plasmalogens in ferroptosis.

Finally, the authors investigated the pathophysiological role of plasmalogen-dependent ferroptosis in disease-related scenarios. Unsurprisingly, human GPX4 knockout cancer cells inoculated into immunodeficient mice initially failed to strive, presumably due to an increase in the ferroptotic demise of the cells. However, after a latency period, mice carrying *GPX4*^*−/*^^−^ cancer cells developed tumors that proliferated as quickly as GPX4-expressing parental cancer cells. Further lipidomics and genetic analysis showed that such *GPX4*^*−/−*^ cells had not reacquired GPX4 expression and instead downregulated the synthesis of plasmalogens to become resistant against ferroptosis. In contrast, the differentiation of neuronal SY5Y precursors or cardiomyocytes derived from human induced pluripotent stem cells was associated with an increased sensitivity to ferroptosis induced by GPX4 inhibition, correlating with an increase in PUFA plasmalogens.

In summary, the results presented by Zou et al. revealed a previously unrecognized role for peroxisome-mediated plasmalogen synthesis in promoting ferroptosis. Like any pioneering studies, the current work raises many interesting questions for the future. What particular advantages do peroxisome-synthesized plasmalogens confer to cells beyond rendering them susceptible to ferroptosis? During the induction of ferroptosis, what structural changes need to occur in peroxisomes? Do peroxisomes contribute to ferroptosis by other mechanisms than those related to lipid synthesis? What is the role of ferroptosis in peroxisome biogenesis disorders, such as Zellweger syndrome? Do cells employ pexophagy, the selective autophagic degradation of peroxisomes, as a strategy to regulate their susceptibility to ferroptosis?
